# Radiation and Contrast Medium Dosage Optimization of Coronary CT Angiography While Combining Personalized Patient Protocol Technology and Automated Tube Voltage Selection Technology

**DOI:** 10.3390/diagnostics16172804

**Published:** 2026-08-31

**Authors:** Jingkun Sun, Yang Zhou, Ge Zhang, Lijun Zhang, Xinnan Shen, Zhiwei Zhang

**Affiliations:** Department of Radiology, The First Affiliated Hospital of Chongqing Medical University, No. 1 Youyi Road, Yuzhong District, Chongqing 400016, China; sunjingkun@hospital.cqmu.edu.cn (J.S.); 202591@hospital.cqmu.edu.cn (Y.Z.); 2h4509@hospital.cqmu.edu.cn (G.Z.); 20h4141@hospital.cqmu.edu.cn (L.Z.);

**Keywords:** coronary computed tomography angiography, personalized patient protocol technology, automated tube voltage selection, contrast media, radiation dosage, image quality

## Abstract

**Objective**: This study aims to investigate whether combining automated tube voltage selection (ATVS) with P3T for contrast injection in coronary computed tomography angiography (CCTA) allows for patient-tailored scanning protocols. Specifically, we assessed whether this combination maintains equivalent image quality and adequate diagnostic image quality. **Methods:** This retrospective study included 378 patients who underwent CCTA using ATVS and P3T technologies. We divided the patients into three groups based on the scanning tube voltage: 70-, 80-, and 90-kV groups. Furthermore, each group was divided into sequence and spiral scanning subgroups based on the acquisition method. The CM injection volume and rate, volume CT dose index (CTDIvol), and dose–length product (DLP) were recorded. The CT value of the enhanced coronary artery, noise, signal-to-noise ratio (SNR), and contrast-to-noise ratio (CNR) were calculated by artery segment. Image quality was evaluated using a four-point grading scale in a double-blind manner. **Results:** The SNR did not differ among the groups in any segments (*p* > 0.05). And the subjective image quality showed only minor differences among the groups. The body weight, which determines ATVS and P3T protocol algorithm, is positively correlated with the automatic tube voltage, CM injection volume, and effective radiation dose. Furthermore, when using the same tube voltage under the combined ATVS and P3T protocols, sequence scanning yielded a lower radiation dose than spiral scanning (*p* < 0.05). **Conclusions:** CCTA combining ATVS and P3T technology enables individualized scanning protocols while maintaining adequate diagnostic image quality.

## 1. Introduction

Coronary computed tomography angiography (CCTA) is a major noninvasive imaging modality for the diagnosis of coronary heart disease [1,2] with a high negative predictive value [3]. It is widely used as a pre-screening or adjunctive examination before invasive catheterization. In patients with stable chest pain, adding CCTA to standard care significantly reduces the 5-year risk of coronary heart disease-related death and non-fatal myocardial infarction [4]. In the elective vascular surgery of asymptomatic patients, all-cause mortality at long term was found to be independently predicted by the total plaque burden measured with CCTA and silent coronary ischemia diagnosed through CT-derived fractional flow reserve [5]. Moreover, there are prognostic data that can be obtained through CCTA in patients who have no serious luminal stenosis on their initial vascular imaging [6]. However, drawbacks such as radiation exposure and iodinated contrast media (CM) injections carry potential risks for cancer and contrast-induced nephropathy [7]. Following the “as low as reasonably achievable” radiation safety principle, researchers aim to reduce radiation doses and iodinated CM while preserving image quality. This study combines these technologies to achieve that goal.

CT is currently the greatest contributor to the radiation burden associated with medical imaging examinations [8]. Applying tube voltage and current adjustment technology can affect the CT radiation and image quality but requires manual optimization for each patient. Meanwhile, CCTA technology improvements, including tube current modulation, prospective electrocardiogram triggering, high-pitch acquisition, and iterative reconstruction techniques, can effectively reduce radiation dose while maintaining diagnostic image quality [9,10]. Furthermore, automated tube voltage selection (ATVS; Siemens Healthcare GmbH, Erlangen, Germany) enables radiation dose reduction by automatically selecting the appropriate tube voltage and modulating tube current based on the patient’s anatomy and examined region as determined by scout-image acquisition [11,12,13,14]. These automated algorithms can optimize the tube voltage and current to reduce radiation exposure while maintaining the diagnostic image quality [15].

On the other hand, iodinated CM can cause contrast-induced nephropathy [16,17]. Consequently, the contrast injection dose should be reduced while preserving diagnostic coronary enhancement for CCTA. Previous studies have revealed that an enhanced CT value of 300–500 HU meets the diagnostic requirements for coronary arteries [18,19]. Appropriate parameter settings to control the CT value are the key to successful CCTA examination, imaging homogenization, and individual scan technologies in clinical practice. To balance the effects of tube voltage and contrast volume on the CT attenuation value, personalized patient protocol software can determine the appropriate CM injection protocol. P3T is a commercial software that provides individualized contrast medium injection protocol. It has been found that the system, based on the concept of Bae and Kalafut et al., who developed it, has a nonlinear relationship between the body weight of the patient and the rate of delivery of iodine to reach the target enhancement [20]. P3T will automatically produce an individualized injection program depending on the body weight of the patient and the concentration of the selected contrast medium in terms of its iodine content. Moreover, when the tube voltage changes due to automated tube voltage selection (ATVS), the software will be able to change the iodine delivery speed in response to the expected amount of enhancement at the chosen kVp, thus ensuring that there is a similar diagnostic attenuation goal among patients [19].

Overall, scanning and CM injection parameters influence coronary contrast enhancement [19,21]. However, few studies have comprehensively evaluated the simultaneous implementation of ATVS and P3T within a unified CCTA acquisition protocol. Consequently, this study aimed to investigate combining ATVS and P3T technologies for CCTA patient-tailored scanning protocols while maintaining satisfactory diagnostic image quality.

## 2. Materials and Methods

### 2.1. Study Population

**Figure 1:**
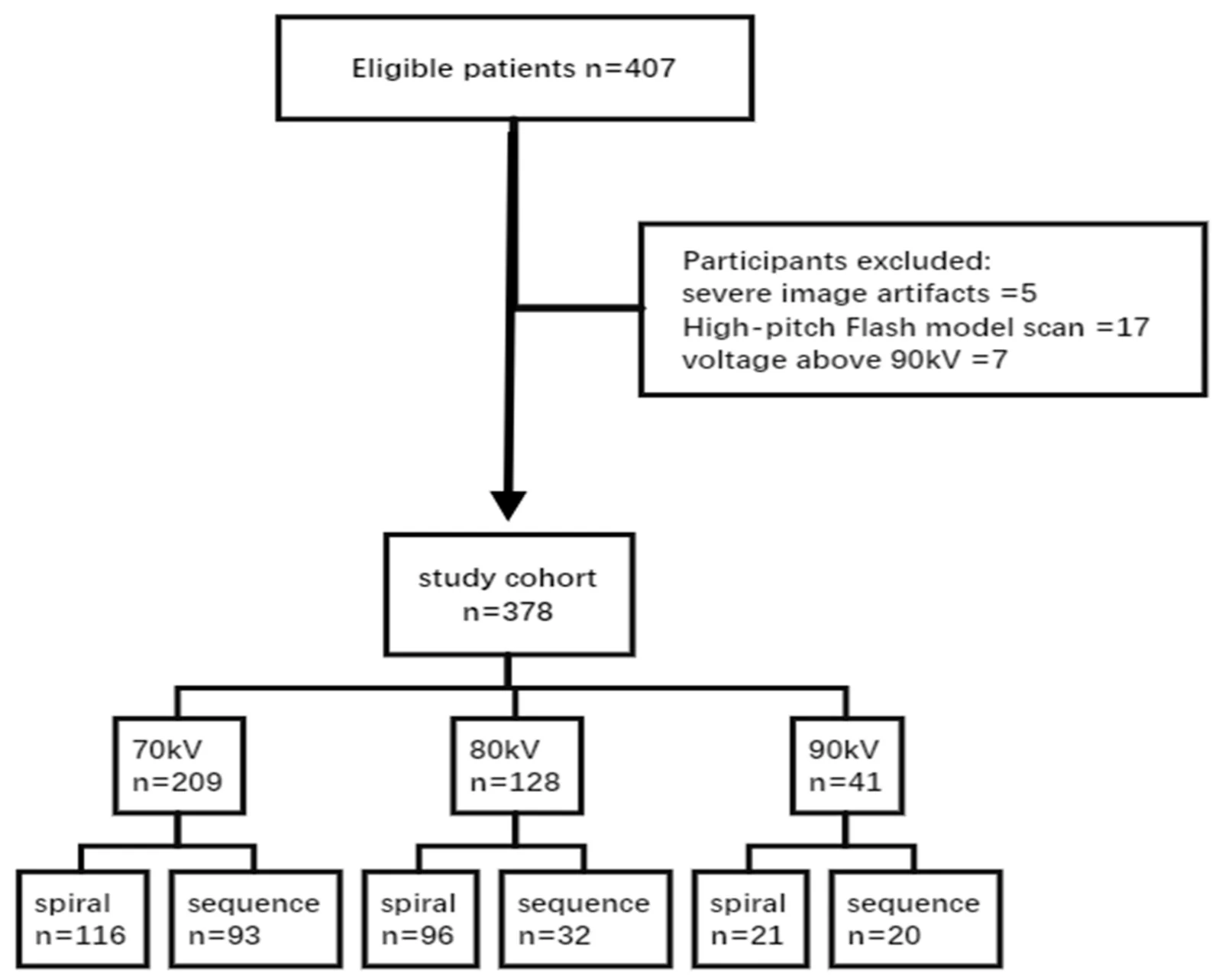
Flowchart of patient selection and grouping.

This retrospective study enrolled 407 patients who underwent CCTA with a third-generation dual-source CT scanner (Somatom Force, Siemens Healthineers, Forchheim, Germany) within one year. None of the patients were given beta-blockers or sublingual nitroglycerin prior to CCTA acquisition. The participants were all subjected to standard breath-hold training (15 s) and detailed pre-examination instructions by specialized nurses prior to scanning. Patients who had atrial fibrillation, severe arrhythmias or severe tachycardia (resting heart rate of 110 beats per min) were not included in the study because irregular heart rhythm interferes with coronary opacification and image quality. After excluding five scans with image artifacts, 17 scans acquired using the large-pitch flash scan mode, and seven scans acquired at a tube voltage > 90 kV, 378 CCTA scans were included in the analysis. Based on the scanning tube voltage, patients were divided into three groups: 70 (*n* = 209), 80 (*n* = 128), and 90 kV (*n* = 41) groups. Furthermore, each group was divided into sequence or spiral scanning subgroups based on the scanning mode (Figure 1). The institutional ethics committee approved the study protocol. The requirement of obtaining informed consent was waived because of the retrospective study design.

### 2.2. CM and Scanning Injection Protocol

The P3T CM injection protocol was given to all patients in Iomeron (400 mgI/mL), and the time of the injection was 12 s. The weight-based factors were 0.5 at 70 kV, 0.4 at 80 kV and 0.375 at 90 kV. The volume/constant injection time of 12 s was used as the flow rate. The personalization principle of P3T has been described previously [22]; because the proprietary weighting formula is not publicly disclosed by the vendor, we report the operational parameters and the resulting dose and flow rate outputs so that our protocol can be reproduced on the same platform.

CCTA was performed using clinically established protocols on a third-generation dual-source CT scanner (Somatom Force, Siemens Healthineers, Forchheim, Germany,). The scanning process was determined by the resting heart rate and breath-hold capacity of the patients. Patients who had a resting heart beat rate of less than 65 beats per minute, good breathing ability, and a normal cardiac rhythm were scanned with the ECG gated sequence. Retrospective ECG-gated spiral scanning was carried out on those patients whose heart rate was above or equal to 80 beats per minute, who did not have a good breathing ability, or who had cardiac arrhythmic. In patients with a resting heart rate of 65 to 79 beats per minute, one of the two options, i.e., either spiral scanning or sequence scanning, could be chosen. Scans were acquired in the craniocaudal direction from the carina to the cardiac base. The scanner was operated in the dual-source mode using a detector collimation of 168–198 × 0.6 mm, z-flying focal spot, and gantry rotation time of 0.25 s. Automated attenuation-based tube current modulation (CARE Dose 4D, Siemens Healthineers) was used with a reference tube current–time product of 340 mAs/rotation at a tube voltage of 100 kV. The tube voltage in ATVS ranged from 70 to 130 kV and was increased at 10 kV intervals. Dedicated bolus-tracking software (CARE Bolus, Siemens Healthineers, Forchheim, Germany, syngo-via platform) was used to monitor contrast enhancement. Within 10 s after CM injection, fluoroscopic monitoring was performed at the level of the descending aorta. When the radiodensity exceeded 60 HU, scanning started after a 7 s delay owing to respiratory control. All scan images were reconstructed with a section thickness of 0.75 mm and an increment of 0.5 mm using a medium-smooth soft-tissue kernel (Bv40) and advanced modeled iterative reconstruction strength 3.

### 2.3. Data Measurement and Evaluation

We retrospectively recorded each patient’s examination number, name, age, height, weight, applied tube voltage, reference tube current–time product, CT dose index, dose–length product, CM dose, and CM flow rate. The original data were transferred to a dedicated post-processing workstation (Syngo.via VB40, Siemens Healthineers) to evaluate the image quality. The effective dose (ED) was estimated by multiplying the dose–length product by a cardiac-specific conversion factor (k = 0.026 mSv/mGy⋅cm) [23]. 

### 2.4. Objective Evaluation of Image Quality

The mean CT value and standard deviation (SD) values were measured by placing a region of interest (ROI) in the aortic root, proximal segments of the right coronary artery (RCA), left anterior descending artery (LAD), left circumflex coronary artery (LCx), and pericardial fat at all levels. ROI was made as large as possible and placed within the contrast-enhanced lumens while carefully avoiding plaques, calcifications, stenosis, and the vessel wall. The signal-to-noise ratio (SNR) was calculated as the attenuation of the relevant artery divided by noise [24]. The contrast-to-noise ratio (CNR) was calculated by dividing the difference between the attenuations of the relevant artery and pericardial fat by the noise in fat tissue. Two experienced technicians performed these measurements, and the average values were used for analyses. 

**Figure 2:**
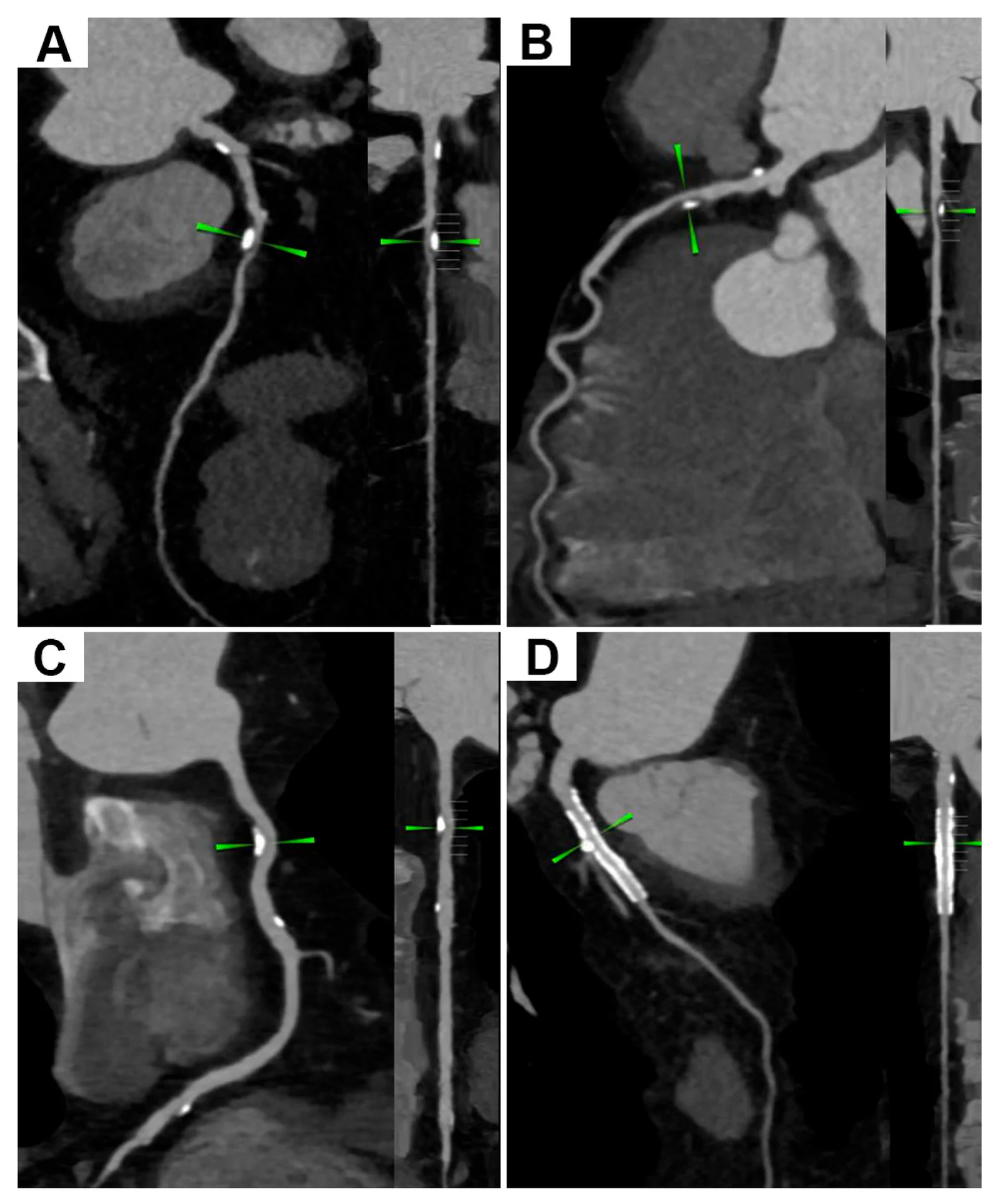
Representative CCTA images showing calcified plaque-derived stenosis and stent appearance under different scanning protocols. (**A**) Calcified plaque with moderate luminal stenosis at 70 kV. (**B**) Mixed plaque with moderate luminal stenosis at 80 kV. (**C**) Calcified plaque with mild luminal stenosis at 90 kV. (**D**) Post-stenting status with patent lumen at 90 kV.

Two doctors with over five years of experience in CCTA diagnostic examinations evaluated the image quality in a double-blinded manner. Discrepancies between the two readers were resolved via consensus or adjudication by a senior third reader. Image quality was graded on a four-point Likert scale. A score of “4” indicated that the coronary vessels were well and uniformly developed and clearly displayed with good contrast. Score “3” indicated that the development of the coronary artery was non-uniform or over-enhanced with clear boundaries and good contrast not affecting the diagnosis. Score “2” indicated that the development of the distal coronary vessels was uneven or light, with moderately blurred boundaries of some vessels and good contrast not affecting the diagnosis. Score “1” indicated that the development of the coronary artery was insufficient or uneven, with poor contrast not meeting the diagnostic image quality. Figure 2 illustrates the visualization of calcified plaque-derived stenosis and stents on CCTA under different scanning conditions.

### 2.5. Statistical Analysis

The distribution of continuous variables was evaluated using the nonparametric Kolmogorov–Smirnov test. Non-normally distributed data were evaluated using the nonparametric Kruskal–Wallis test. Data are expressed as the median (interquartile range). Spearman’s rank-order correlation analysis was used to evaluate bivariate associations among body weight, tube voltage, CM volume, and ED. Multiple linear regression was performed to identify factors associated with ED, CM volume, CNRmean, and SNRmean, with body weight, tube voltage setting, scan mode, and age included as covariates in the models. Multicollinearity was assessed by variance inflation factors (VIFs). Model fit of each regression model was described using R^2^ and reported adjusted R^2^. Statistical analyses were performed using Statistical Package for the Social Sciences software (version 26.0; SPSS Inc; Chicago, IL, USA). Two-sided *p*-values < 0.05 were considered to be statistically significant.

## 3. Results

### 3.1. Objective Image Quality

Table 1 lists the objectively evaluated image quality values in 70, 80, and 90 kV groups. The SNR did not differ significantly among the groups. CNR differed significantly only in the LCx and RCA. With increasing tube voltage, CT values of the aortic root, LAD, and LCx branch decreased statistically, whereas the CT attenuation value in the right coronary artery decreased without reaching statistical significance.

Table 2 summarizes the objectively assessed image quality values for the spiral and sequence scanning subgroups. No significant differences in SNR or CNR were identified between the two subgroups across all voltage cohorts. In the 90 kV cohort, CT values of the left anterior descending artery, left circumflex artery, and right coronary artery were significantly higher in the spiral scanning subgroup than in the sequential scanning subgroup. In contrast, in the 70 and 80 kV cohorts, a significant difference in CT attenuation was observed only in the RCA, with higher values in the spiral scanning subgroup than in the sequential scanning subgroup.

### 3.2. Subjective Image Quality

Subjective image quality showed only minor differences among the groups. After the consistency test, Cohen’s kappa of the two raters was >0.6, indicating strong consistency. The score of all vessels was ≥2. Table 3 presents the subjective image quality scores in the 70, 80, and 90 kV groups. Of note, only the aortic score from observer 2 (OB2) produced a nominal *p* value of 0.022. Given that this represented an isolated positive finding across multiple subjective comparisons, and median scores were comparable across groups, this difference is likely of limited clinical relevance.

### 3.3. Contrast Medium Volume

CM injection parameters were selected using P3T. As the patient’s weight increased, the tube voltage, CM volume, and injection rate increased. In the 70, 80, and 90 kV groups, the CM volume were 29 (28–31), 34 (32–36), and 39 (36–42) mL, respectively. In addition, the CM injection rates were 2.4 (2.3–2.6), 2.8 (2.7–3.0), and 3.3 (3.0–3.5) mL/s, respectively, both indicating significant differences. Table 4 presents the CM volume and injection rate in each group and subgroup.

### 3.4. Radiation Dose

The radiation dose increased with increasing tube voltage. EDs in the 70, 80, and 90 kV groups were 3.5 (2.5–4.8), 6.9 (5.3–8.4), and 9.0 (5.4–11.9) mSv, respectively, indicating significant differences (*p* < 0.05). Table 5 presents the radiation dose using ATVS in each group.

Table 6 displays the radiation and CM volume in each subgroup of each group. The radiation dose was significantly higher in the spiral scanning subgroup than in the sequence scanning subgroup in each group. The CM volume was higher in the spiral scanning subgroup than in the sequence scanning subgroup in each group, although the differences were not statistically significant.

### 3.5. Bivariate Correlations

Bivariate correlations between the clinical and dosimetric variables are presented in Table 7. Significant positive correlations were observed among all assessed parameters. Body weight was positively correlated with automatic tube voltage, CM volume, and ED. Automatic tube voltage revealed strong positive correlations with CM volume and ED. In addition, CM volume was positively correlated with ED.

### 3.6. Multiple Linear Regressions

Multivariable linear regression analyses were performed to identify variables that showed independent statistical associations with effective dose, CM volume, mean CNR, and mean SNR within the mode, and the corresponding results are presented in Table 8. Body weight, tube voltage (80 and 90 kV versus 70 kV), scan mode, and age represented significant predictors showing independent statistical associations with ED. Higher body weight was associated with increased ED (*B* = 0.061, β = 0.228, *p* < 0.001). Compared with 70 kV, both 80 kV (*B* = 2.201, β = 0.351, *p* < 0.001) and 90 kV (*B* = 4.570, β = 0.479, *p* < 0.001) were positively correlated with ED. Sequence scan revealed a negative association with ED (*B* = −2.883, β = −0.473, *p* < 0.001). Older age was linked to slightly higher ED (*B* = 0.025, β = 0.096, *p* = 0.001). Body weight, tube voltage, and scan mode were significant predictors showing independent statistical associations with CM volume. Body weight showed the strongest association with CM volume, with a standardized coefficient of 0.651, and each 1 kg increase in weight corresponded to an increase of 0.265 in CM volume. Compared with 70 kV, scanning at 80 and 90 kV was associated with increases in CM volume of 2.438 and 5.837, respectively. Sequence scanning showed a negative association with CM volume, with a small yet statistically significant effect in this multivariable model. In contrast, age revealed no significant independent influence on CM volume (*p* = 0.626). Only weight had an independent statistical association with mean CNR (*B* = −0.085, β = −0.148, *p* = 0.013) and mean SNR (*B* = −0.064, β = −0.140, *p* = 0.019). Notably, these multivariable models demonstrate independent statistical associations within the study cohort rather than causal effects of tube voltage, which was selected automatically according to patient body habitus.

## 4. Discussion

The ATVS and P3T injection protocols of the individualized CM have been studied in the past, and a number of researchers have analyzed the optimization of the protocol with these technologies. It has been shown by Mangold et al. [25] that ATVS allows for adjusting the tube voltage individually in CCTA on the third-generation dual-source CT system, which will significantly decrease the radiation dose without compromising the quality of images used for diagnostic purposes. Jayamani et al. concluded that P3T software for contrast injection in coronary CT angiography effectively provides sufficient diagnostic coronary attenuation (more than 350 HU) in all weight groups but does not compromise diagnostic image quality [26]. However, most previous studies have focused on lowering radiation exposure or reducing CM volume separately. Few studies have directly compared 70, 80, and 90 kV scanning protocols under a unified workflow with ATVS and P3T implemented simultaneously. In the present study, we comprehensively evaluated the trade-offs between radiation exposure, contrast medium dosage and objective image quality across distinct tube voltage subgroups. Furthermore, multivariate linear regression analyses were performed to identify factors independently associated with these outcome measures. The analysis provided quantitative evidence to support this combined scanning strategy.

Radiation and CM doses should be reduced in CCTA because of the risk of complications. Reduction in tube voltage can significantly decrease the radiation dose [27], which is consistent with the findings of this study. The conventional tube voltage for coronary artery scanning is 120 kV [28]. The ATVS enables patient-specific selection of tube voltage based on patient characteristics and scanning conditions, thereby reducing radiation exposure. The tube voltage can be reduced to as low as 70 kV. This study demonstrated that the application of ATVS enables the tailored selection of an optimal tube voltage according to individual patient characteristics while preserving diagnostic image quality. Lowering the tube voltage in CCTA results in relatively higher attenuation of iodine and thus higher vascular attenuation when intravascular CM is administered [29]. This effect can be used to reduce the intravenously administered CM dose. The iodine concentration of CM is also relevant. Although a low tube voltage increases image noise, we used a high iodine concentration of CM for the blood vessels to obtain a high iodine signal at a low tube voltage and maintain a certain SNR. Moreover, a high iodine concentration of CM can reduce the flow rate and volume of CM injection and the risk of CM leakage. These factors make it difficult to formulate a CM protocol. However, P3T resolves this issue. In routine CCTA examinations, the contrast medium volume ranges from 50 to 120 mL, with an injection rate of 5–7 mL/s [28]. In the present study, the corresponding values were 29 (28–31) and 2.4 (2.3–2.6) mL/s at 70 kV, 34 (32–36) and 2.8 (2.7–3.0) mL/s at 80 kV, and 39 (36–42) and 3.3 (3.0–3.5) mL/s at 90 kV, respectively. Furthermore, a single 50 mL vial of 400 mg I/mL CM was uniformly used for all examinations, effectively eliminating CM waste.

The best enhancement value of diagnostic CCTA images is 300–500 HU, which we considered acceptable for the aortic root in this study. A higher CT value of the coronary artery leads to significant underestimation of stenosis in smaller vessels, whereas a lower CT value results in overestimation [19,30]. In this study, by objectively evaluating image quality, the median CT value of CCTA images fell within this range, revealing that the image enhancement obtained in the study met the diagnostic image quality. The subjective evaluation score was ≥2 points, indicating that P3T and ATVS met the imaging diagnostic image quality. SNR did not differ among the groups, indicating good consistency of image quality. CT attenuation reduction with increasing tube voltage was statistically significant in the aortic root, LAD and LCx, but not in the right coronary artery. The RCA nonetheless showed the same directional decline. This non-significant result is most plausibly explained by two RCA-specific factors. First, the RCA has the greatest inter-individual anatomical variability of the three coronary arteries: it originates from the right aortic sinus and runs in the right atrioventricular groove, where its course, the amount of surrounding epicardial fat, and the proximal lumen caliber vary considerably between patients. Additionally, because the proximal RCA is often oriented obliquely to the axial acquisition planes, ROI placement is more variable and partial-volume averaging is more pronounced. Second, the RCA lies along the right heart border and is especially susceptible to cardiac-motion-induced blurring, which raises the standard deviation of repeated attenuation measurements. The resulting wider between-subject variance enlarges the confidence intervals around the group means and reduces the statistical power to detect the tube voltage effect. The directional decrease in RCA attenuation is therefore consistent with, rather than contradictory to, the significant reductions observed at the aortic root, LAD, and LCx. However, the radiation dose was significantly lower in the sequential scanning group than in the spiral scanning group at the same tube voltage, without a significant difference in image quality. CT values of the left anterior descending branch, left circumflex branch, and RCA were higher in the spiral scanning subgroup than in the sequential scanning subgroup in the 90 kV group, whereas only the RCA revealed significantly higher attenuation in the spiral scanning subgroup of the 70 and 80 kV groups. The average scanning time of spiral scanning was 2.4 s, while that of sequential scanning was 3.5 s. Extended scanning time could affect the CT attenuation of some vessels. Accordingly, the scanning mode should be reasonably selected.

The combined correlation and regression analyses revealed distinctly influencing patterns for dose-related and image quality outcomes. In bivariate analysis, body weight, tube voltage, and dose metrics were closely correlated with each other. Multivariable regression showed that tube voltage, scan mode, and body weight were significant predictors showing independent statistical associations with effective radiation dose and CM volume. However, only body weight exhibited an independent statistical association with mean CNR and mean SNR after covariate adjustment. This discrepancy can be largely explained by the clinical automatic scanning protocol used in this study. As tube voltage was automatically selected according to patient body habitus, heavier patients tended to receive higher kV settings, resulting in unbalanced weight distribution across subgroups. This inherent confounding contributed to the apparent crude association between tube voltage and image quality. After adjusting for body weight, the association between tube voltage and CNR or SNR was no longer significant. In addition, automatic exposure control adaptively regulates mAs and kilovoltage variation, which counteracts theoretical changes in noise and signal.

In addition to dose and contrast optimization, clinical interpretation of CCTA in the populations that we are targeting with our low-dose strategy should be highlighted. Calcified plaques are susceptible to blooming artifact, where beam-hardening and partial-volume effects cause calcification to appear enlarged and can exaggerate stenosis severity. This effect is most pronounced in older patients who tend to have extensive coronary calcification. Lowering tube voltage increases iodine attenuation and helps maintain vascular contrast at a reduced contrast medium dose, but it also widens the attenuation gap between calcium and soft tissue and can raise image noise, which may aggravate blooming. The high iodine concentration (Iomeron 400 mg I/mL) and iterative reconstruction (ADMIRE, strength 3) used here help preserve the signal-to-noise ratio, yet blooming remains an inherent limitation of CCTA that must be weighed when triaging patients to invasive angiography.

The safety of repeated CCTA is an additional consideration that our protocol directly addresses. Patients with known or suspected coronary artery disease frequently require several contrast-enhanced CT examinations, either for surveillance or because an initial study is indeterminate, and these repeat studies accumulate effective radiation dose and iodinated contrast medium exposure. Our individualized low-dose approach, which couples automatic tube voltage selection down to 70 kV with sequence scanning (median effective dose of 3.5 mSv at 70 kV), thereby mitigates the cumulative burden for patients who need repeated imaging over time.

CCTA is frequently used in the elderly with multiple comorbidities instead of invasive coronary angiography, which is a less invasive and safer first-line test [31]; however, the choice between CCTA and invasive angiography remains actively debated [31]. Old age also implies more severe calcification (worsening blooming), more renal comorbidity (increasing the risk of contrast-induced nephropathy) and more frequent arrhythmias, so CCTA should be applied judiciously in this population. Elderly patients commonly carry multi-arterial atherosclerotic burden and multiple comorbidities that complicate management [32]. The combined ATVS and P3T strategy is particularly suited to these vulnerable patients, provided that careful patient selection is maintained.

This study has some limitations. First, only tube voltages of 70, 80, and 90 kV were analyzed due to insufficient data for images obtained above 90 kV. Since ATVS automatically selects kilovoltage based on patient body habitus, this may limit generalizability to overweight and obese populations. Second, this study did not assess the diagnostic performance for calcified plaques and other lesions, nor did it carry out comparative analyses with invasive coronary angiography. Consequently, the impact of our findings on imaging diagnosis remains limited. Third, images acquired in the high-pitch turbo flash scan mode were not analyzed because of different P3T parameters. Fourth, the study design was retrospective. The number of cases varied across groups, and only 400 mg I/mL of CM was analyzed. We aimed to overcome these limitations in follow-up research.

To combine ATVS and P3T, the tube voltage, CM volume, and injection rate should be reasonably selected. If conditions permit, sequence scanning should be selected to reduce the radiation dose. Our study indicates that ATVS combined with P3T allows individualized scanning of patients undergoing CCTA while maintaining adequate diagnostic image quality. The combination of these two technologies can achieve comparable image quality across tube voltage subgroups, demonstrating the feasibility and technical performance of the combined ATVS–P3T strategy, rather than proven superiority or enhanced clinical diagnostic performance.

## Figures and Tables

**Table 1:** The objective image values in the 70-, 80-, and 90-kV groups.

Tube Voltage (kV)	No. of Patients		CT Value (HU)				SD				SNR			CNR		
			Aorta	LAD	LCx	RCA	Aorta	LAD	LCx	RCA	LAD	LCx	RCA	LAD	LCx	RCA
70	209	Median	461.1	422.9	371.1	397.5	30.4	24.8	23.7	23.5	17.2	15.7	16.6	22.2	21.4	21.7
		IQR	407.6–511.4	371.3–475.2	308.0–431.3	322.4–466.3	27.7–34.8	18.9–30.7	18.4–29.8	18.6–30.3	13.5–22.5	11.6–20.4	12.2–21.4	18.1–29.2	16.1–27.2	16.7–27.9
80	128	Median	423.6	380.5	329.4	398.2	27.0	23.2	23.9	27.3	16.1	14.3	14.9	21.4	19.4	19.8
		IQR	386.2–473.9	338.8–418.7	286.0–374.9	332.8–450.4	23.6–31.0	18.0–28.7	17.8–30.2	20.0–33.2	12.9–21.7	10.2–18.3	11.5–19.5	17.2–28.6	14.8–24.8	15.2–25.4
90	41	Median	407.5	366.1	324.7	366.7	24.0	22.2	20.3	24.4	17.1	16.4	13.9	22.7	21.5	18.6
		IQR	358.4–461.7	317.1–409.5	276.4–370.6	269.3–433.3	22.2–29.4	15.8–29.5	15.2–25.4	18.9–32.0	12.2–23.1	12.6–21.6	10.8–18.8	16–31.5	17.4–29.6	15–24.5
*p*-value			0.001	0.001	0.001	0.183	0.001	0.119	0.008	0.059	0.691	0.190	0.073	0.752	0.032	0.035

**Table 2:** Objective evaluation index results of image quality in each subgroup.

Tube Voltage (kV)	Protocol	No. of Patients		CT Value (HU)				SD				SNR			CNR		
				Aorta	LAD	LCx	RCA	Aorta	LAD	LCx	RCA	LAD	LCx	RCA	LAD	LCx	RCA
70	Spiral	116	Median	461.6	424.8	367.5	431.4	29.0	25.8	24.2	24.9	17.1	15.5	16.9	22.4	20.5	22.5
			IQR	407.4–512.2	376.9–472.7	302.7–432.3	360.3–484.1	26.5–32.3	18.9–31.3	18.5–30.0	19.3–30.9	13.1–22.0	11.6–19.5	12.3–21.7	18.0–28.4	16.2–25.7	17.2–27.9
	Sequence	93	Median	460.7	410.1	371.3	358.9	33.1	24.7	22.9	22.6	17.3	16.5	15.9	22.2	22.4	21.0
			IQR	407.9–510.6	367.2–479.1	312.9–431.3	297.8–415.7	29.9–36.7	19.0–30.0	18.0–28.9	17.4–29.6	13.6–22.8	11.8–21.0	11.4–20.4	18.1–29.7	16.0–28.5	16.4–28.2
	*p*-value			0.609	0.878	0.875	<0.001	<0.001	0.159	0.325	0.232	0.618	0.281	0.289	0.683	0.243	0.725
80	Spiral	96	Median	424.6	384.4	331.9	411.5	26.4	23.2	25.0	28.6	16.0	13.8	14.9	21.3	19.0	19.4
			IQR	389.6–474.5	346.3–417.7	284.3–379.5	359.5–461.6	23.4–30.9	19.1–28.9	18.4–31.2	21.5–33.6	13.3–21.1	10.2–18.3	11.1–19.5	17.4–28.5	14.5–24.4	14.9–25.3
	Sequence	32	Median	423.0	365.3	324.5	332.4	29.1	23.0	22.3	22.9	19.2	15.0	15.0	25.0	21.2	20.1
			IQR	370.6–468.0	332.5–431.9	287.0–372.6	304.0–408.7	25.2–31.8	16.5–28.2	17.2–27.6	19.3–30.2	12.7–22.5	10.7–18.5	12.5–19.5	16.8–29.1	15.4–25.8	16.6–26.1
	*p*-value			0.605	0.547	0.817	0.001	0.080	0.337	0.192	0.041	0.368	0.562	0.714	0.380	0.495	0.857
90	Spiral	21	Median	430.3	402.7	355.7	410.1	23.8	22.8	21.6	25.0	16.2	14.5	16.9	23.0	19.1	22.6
			IQR	382.7–484.8	333.2–467.5	306.7–396.9	346.4–473.4	21.8–28.8	17.7–30.3	18.4–26.9	20.5–34.8	12.1–22.9	12.5–20.8	11.1–19.9	16.0–30.0	17.5–26.5	15.1–25.9
	Sequence	20	Median	377.2	338.0	287.9	344.8	26.0	22.0	16.7	21.3	17.3	17.2	12.9	22.3	23.7	18.5
			IQR	355.4–447.0	297.6–384.5	260.1–329.7	234.3–388.3	22.8–29.9	12.7–28.9	12.4–22.9	17.9–29.9	12.4–23.3	12.4–22.4	10.4–16.6	16.1–33.8	17.3–32.9	14.9–23.1
	*p*-value			0.110	0.035	0.025	0.008	0.769	0.271	0.030	0.131	0.916	0.424	0.628	0.737	0.242	0.909

**Table 3:** Subjective evaluation index results of image quality in each group.

Tube Voltage (kV)	Observers	Median (IQR)				
		Aorta	LAD	LCx	RCA	Overall
70	OB1	4 (3–4)	4 (3–4)	4 (3–4)	4 (3–4)	4 (3–4)
	OB2	4 (3–4)	4 (3–4)	4 (3–4)	4 (3–4)	4 (3–4)
	k	0.89	0.82	0.82	0.87	0.79
80	OB1	4 (3–4)	4 (3–4)	4 (3–4)	4 (3–4)	4 (3–4)
	OB2	4 (3–4)	4 (3–4)	4 (3–4)	4 (3–4)	4 (3–4)
	k	0.82	0.85	0.77	0.84	0.81
90	OB1	4 (3–4)	4 (3–4)	4 (3–4)	4 (3–4)	4 (3–4)
	OB2	4 (3–4)	4 (3–4)	4 (3–4)	4 (3–4)	4 (3–4)
	k	0.68	1.00	0.95	0.85	0.78
*p*-value	OB1	0.274	0.505	0.095	0.568	0.490
	OB2	0.022	0.270	0.133	0.110	0.112

OB1 and OB2 denote two independent observers. k represents Cohen’s kappa coefficient for inter-observer agreement.

**Table 4:** Contrast media injection parameters in each group.

	70 kV	80 kV	90 kV	*p*-Value
No. of patients	209	128	41	
Age (years)	61 (51–69)	57 (48–65)	57 (50.0–66.5)	0.0386
Weight (kg)	60 (52.5–65.0)	66 (61.3–75.0)	73 (63.5–80.5)	<0.001
CM volume (mL)	29 (28–31)	34 (32–36)	39 (36–42)	<0.001
Flow rate (mL/s)	2.4 (2.3–2.6)	2.8 (2.7–3.0)	3.3 (3.0–3.5)	<0.001

**Table 5:** Radiation dose parameters in each group.

Tube Voltage (kV)	No. of Patients	CTDI_vol_ (mGy)	DLP (mGy⋅cm)	Effective Dose (mSv)
70	209	9.6 (7.4–12.1)	134.2 (96.4–184.7)	3.5 (2.5–4.8)
80	128	17.6 (14.8–20.9)	264.6 (204.3–324.7)	6.9 (5.3–8.4)
90	41	23.4 (18.8–28.8)	346.0 (206.9–456.2)	9.0 (5.4–11.9)
*p*-value		<0.001	<0.001	<0.001

**Table 6:** Radiation and contrast media doses in the sequence and spiral scanning subgroups.

Tube Voltage (kV)	Protocol	No. of Patients		Age (Years)	Weight (kg)	CTDI_vol_ (mGy)	DLP (mGy⋅cm)	ED (mSv)	CM Volume (mL)	Flow Rate (mL/s)
70	Spiral	116	Median	63	60	11.1	174.2	4.5	29.0	2.4
			IQR	54.3–71	52.3–65.0	9.6–13.4	151.7–203.9	3.9–5.3	28–31	2.3–2.6
	Sequence	93	Median	58	60	8	92	2.4	29.0	2.4
			IQR	48.5–66.5	52.5–65	6.4–9.2	77.8–113.1	2–2.9	28.0–30.0	2.3–2.5
	*p*-value			0.003	0.702	<0.001	<0.001	<0.001	0.165	0.165
80	Spiral	96	Median	57	66	19	288.8	7.5	35.0	2.9
			IQR	48–66	62–76	16.3–22.4	247.4–341.1	6.4–8.9	32–36	2.7–3.0
	Sequence	32	Median	58	66	14.2	160	4.2	33.0	2.8
			IQR	50–64	60.3–70	12.9–15.7	137.4–185.8	3.6–4.8	32.0–34.8	2.7–2.9
	*p*-value			0.550	0.932	<0.001	<0.001	<0.001	0.130	0.130
90	Spiral	21	Median	61	76	27.5	426.2	11.1	40.0	3.3
			IQR	49–67.5	63–84	24.2–31.7	369.3–535.0	9.6–13.9	36.5–44	3–3.7
	Sequence	20	Median	54.5	73	19	207	5.4	38.0	3.2
			IQR	50.3–65.3	65.3–80.0	15.8–21.3	174.3–238.7	4.5–6.2	36.0–41	3–3.4
	*p*-value			0.250	0.773	<0.001	<0.001	<0.001	0.317	0.317

**Table 7:** Spearman’s rank-order correlations between study variables.

	Weight–Tube Voltage	Weight–CM Volume	Weight–ED	Tube Voltage–CM Volume	Tube Voltage–ED	CM Volume–ED
Rho (ρ)	0.485	0.814	0.464	0.757	0.672	0.652
*p*-value	<0.001	<0.001	<0.001	<0.001	<0.001	<0.001

Rho (ρ) represents Spearman correlation coefficient.

**Table 8:** Multivariable linear regressions for ED, CM volume, mean CNR and mean SNR.

Variables	ED			CM Volume			Mean CNR			Mean SNR		
	*B* (95%CI)	Beta	*p*-Value	*B* (95%CI)	Beta	*p*-Value	*B* (95%CI)	Beta	*p*-Value	*B* (95%CI)	Beta	*p*-Value
Constant	2.772 (1.142, 4.403)	-	0.001	15.136 (13.607, 16.665)	-	<0.001	26.139 (19.880, 32.397)	-	<0.001	19.448 (14.474, 24.422)	-	<0.001
Weight (kg)	0.061 (0.044, 0.079)	0.228	<0.001	0.265 (0.248, 0.281)	0.651	<0.001	−0.085 (−0.152, −0.018)	−0.148	0.013	−0.064 (−0.118, −0.011)	−0.140	0.019
80 kV (vs. 70 kV)	2.201 (1.802, 2.600)	0.351	<0.001	2.438 (2.064, 2.812)	0.257	<0.001	−0.690 (−2.220, 0.841)	−0.052	0.376	−0.670 (−1.887, 0.547)	−0.063	0.280
90 kV (vs. 70 kV)	4.570 (3.966, 5.174)	0.479	<0.001	5.837 (5.271, 6.404)	0.405	<0.001	0.708 (−1.610, 3.027)	0.035	0.548	0.504 (−1.339, 2.347)	0.031	0.591
Scan mode	−2.883 (−3.233, −2.533)	−0.473	<0.001	−0.774 (−1.102, −0.445)	−0.084	<0.001	0.986 (−0.357, 2.330)	0.076	0.150	0.441 (−0.627, 1.509)	0.043	0.417
Age (years)	0.025 (0.010, 0.039)	0.096	0.001	−0.003 (−0.017, 0.010)	−0.009	0.626	0.023 (−0.034, 0.079)	0.042	0.427	0.028 (−0.016, 0.073)	0.065	0.215

*B* represents the unstandardized regression coefficient, CI denotes the confidence interval, and Beta indicates the standardized regression coefficient. All predictors yielded variance inflation factor (VIF) values between 1.067 and 1.373, which ruled out obvious multicollinearity. The adjusted R^2^ was 0.699 for effective dose, 0.884 for CM volume, and 0.027 for both mean CNR and mean SNR.

## Data Availability

The datasets used and/or analyzed during the current study are available from the corresponding author on reasonable request.
